# Definitions, components and processes of data harmonisation in healthcare: a scoping review

**DOI:** 10.1186/s12911-020-01218-7

**Published:** 2020-09-14

**Authors:** Bey-Marrié Schmidt, Christopher J. Colvin, Ameer Hohlfeld, Natalie Leon

**Affiliations:** 1 Cochrane South Africa, South African Medical Research Council, Francie Van Zijl Dr, Parow Valley, Cape Town, 7501 South Africa; 2 Division of Social and Behavioural Sciences, School of Public Health and Family Medicine, University of Cape Town, Cape Town, South Africa; 3 Department of Public Health Sciences, University of Virginia, Charlottesville, USA; 4 Department of Epidemiology, School of Public Health, Brown University, Providence, USA; 5 Health Systems Research Unit, South African Medical Research Council, Cape Town, South Africa

**Keywords:** Data harmonisation, Health information exchange, Health information system, Scoping review

## Abstract

**Background:**

Data harmonisation (DH) has emerged amongst health managers, information technology specialists and researchers as an important intervention for routine health information systems (RHISs). It is important to understand what DH is, how it is defined and conceptualised, and how it can lead to better health management decision-making. This scoping review identifies a range of definitions for DH, its characteristics (in terms of key components and processes), and common explanations of the relationship between DH and health management decision-making.

**Methods:**

This scoping review identified relevant studies from 2000 onwards (date filter), written in English and published in PubMed, Web of Science and CINAHL. Two reviewers independently screened records for potential inclusion for the abstract and full-text screening stages. One reviewer did the data extraction, analysis and synthesis, with built-in reliability checks from the rest of the team. We developed a narrative synthesis of definitions and explanations of the relationship between DH and health management decision-making.

**Results:**

We sampled 61 of 181 included to synthesis definitions and concepts of DH in detail. We identified six common terms for data harmonisation: record linkage, data linkage, data warehousing, data sharing, data interoperability and health information exchange. We also identified nine key components of data harmonisation: DH involves (a) a process of multiple steps; (b) integrating, harmonising and bringing together different databases (c) two or more databases; (d) electronic data; (e) pooling data using unique patient identifiers; and (f) different types of data; (g) data found within and across different departments and institutions at facility, district, regional and national levels; (h) different types of technical activities; (i) has a specific scope. The relationship between DH and health management decision-making is not well-described in the literature. Several studies mentioned health providers’ concerns about data completeness, data quality, terminology and coding of data elements as barriers to data utilisation for clinical decision-making.

**Conclusion:**

To our knowledge, this scoping review was the first to synthesise definitions and concepts of DH and address the causal relationship between DH and health management decision-making. Future research is required to assess the effectiveness of data harmonisation on health management decision-making.

## Background

Data harmonisation (DH) in healthcare is a digital, technology-based innovation that can potentially help routine health information systems (RHISs) function at their best. It can help organise and integrate large databases containing routine health information [1]. Designing, developing and implementing DH interventions has the potential to strengthen aspects of the health system, by enhancing RHISs to high-quality and relevant information that can support decisions, actions and changes across all components and levels of the health system [2, 3]. When RHISs are functioning properly, they can help health practitioners and managers identify and close gaps in health service delivery as well as inform their planning, implementation and monitoring of interventions [4, 5]. They can also help deal address problems related to using different variables and indicators for collecting, analysing and reporting health information across programmes [6], which is common in low-and-middle-income (LMIC) settings. Other challenges to effective RHIS functioning include the production of poor-quality data that cannot easily be exchanged and programmatic fragmentation across levels of the health system, which can result in the duplication and excessive production of data [7].

Lack of standardised data production processes, fragmentation of databases, and errors and duplication in data production are only some of the challenges of RHISs, which may, at first glance be categorised as technical challenges [3, 8]. Solutions to such apparently technical challenges include introducing new data forms, setting up warning systems to detect potential errors, and developing algorithms for integrating different databases.

However, DH interventions for RHISs may not be used effectively if data production and utilisation processes are viewed as merely technical. Given that RHISs are embedded in complex health systems, DH interventions to improve RHIS functions are also influenced by the broader setting, in which dynamic and complex social and technical factors interact [9–11]. There is a need to consider the influence of social factors as well. These may include people’s competencies in dealing with new data production processes, institutional values about data utilisation, and existing relationships between data producers and decision-makers [8, 12, 13].

There is growing recognition that the development and implementation of DH interventions occurs in multiple technical and social contexts, and that DH interventions may differ in definition, purpose and intended outcomes [14]. So, various terms are used for interventions with similar aims and activities to data harmonisation. For example, terms such as record linkage, data warehousing, data sharing and health information exchange are all used to describe data harmonisation-type activities [15–17]; and it is not always clear to which extent these efforts are similar in practice, scope and relevance. The use of multiple terms may not be a problem in itself, but a common understanding of the components and processes will bring more clarity about what constitutes ‘data harmonisation’, and will make it easier to compare and appraise the relevance and usefulness of DH interventions across settings.

Although DH has the potential to enhance RHISs, it is still unclear whether or how it affects health management decision-making. In some cases, DH interventions may not directly impact on improved management decision-making, especially when interventions are more focused on the technical aspects of data production and less on the organisational and behavioural aspects of data use for decision-making [18]. The scope of this review is to therefore understand the different ways in which DH is defined, to identify its components and processes, and to describe whether or how DH can affect health management decision-making. Greater clarity about the range of definitions, components and processes of DH interventions, and its intended outcomes can help to better evaluate its relevance, usefulness, and impact [12].

## Methods

This scoping review was conducted according to the methods outlined by Arksey and O’Malley [19]. They recommend a process that is “not linear but, requiring researchers to engage with each stage in a reflexive way” to achieve both ‘in-depth and broad’ results. This review followed the standard steps for systematic reviews: identifying the research question, identifying relevant studies, selecting studies for inclusion, data extraction and data synthesis. These are detailed in our published study protocol [20].

### Study objectives

This scoping review appraised the definitions, components and processes of data harmonisation activities, and provided a broad explanation of the relationship between data harmonisation interventions and health management decision-making. The specific objectives are:

1. To identify and synthesise the various definitions, components and processes of data harmonisation in healthcare; and
2. To describe the relationship between data harmonisation interventions and health management decision-making.

We took a stepped approach in addressing these objectives. All included studies were used to address Objective 1. To address Objective 2, we sampled studies that were using alternative terms for DH interventions and used those to identify, synthesise and compare similarities and differences in definitions. While executing Objective 1 and 2, we identified a smaller number of studies that contributed to Objective 3.

### Identifying relevant studies

#### Eligibility criteria

Peer-reviewed studies and grey literature were considered eligible for inclusion into the scoping review if they provided a definition or description of DH, and or, a more detailed conceptual explanation (in the form of a model, framework or process) of a DH intervention. Additionally, studies were eligible if they provided an explanation of the causal relationship between DH and health management decision-making (such as through improved quality and accessibility of harmonised information for management and/or the utilisation of harmonised health information for management decision-making). We considered any studies concerned with different technical activities of DH (such as linking, merging, cleaning and transferring). After screening, only studies for which we could access full-text articles were eligible for inclusion in the review.

#### Search strategy

A systematic literature search was conducted in PubMed, CINAHL and Web of Science for eligible studies from 1 January 2000 to 30 September 2018. We limited our search to the year 2000 as digital technology-based innovations began during this period (such as health information exchange) began in high-income countries (predominantly in the United States of America) and when researchers and health system managers in LMICs became interested in the integration of large digital databases [21]. We present the search strategy in the study protocol [20]. Based on preliminary searches we anticipated that these databases would yield the highest results. The search strategies include a combination of keywords and Medical Subject Headings (MeSH) terms related to data harmonisation (concept A) and health information system (concept B). There were no geographic restrictions, but for logistical reasons of time and resources, we only searched for English studies.

### Selecting studies for inclusion

#### Screening records

The first reviewer (BS) conducted all the searches with the help of a librarian and collated the records in the EndNote reference management programme where duplicates were removed. Two reviewers (BS) and second reviewer (AH) then independently screened the records (titles and abstracts) to assess eligibility for full-text review. BS and AH resolved conflicts that emerged at this stage by talking through the inclusion criteria and arriving at a joint decision.

The full-texts of potentially eligible studies were retrieved and assessed by the two reviewers (BS and AH). Final inclusion into the review was based on whether at a minimum the study had a definition or description of a DH intervention or referred to its relationship with health management decision-making. The first reviewer read all full-texts and the second reviewer only read a sample (roughly a third) of the full-texts to verify the first reviewer’s decision about inclusion. BS and AH disagreed on four studies, and after discussion, agreed to exclude the studies.

After finalising screening, the two reviewers then mapped out the characteristics of included studies in an Excel spreadsheet. They recorded the name of the first author, the date, the type of study (primary, review, conceptual, commentary), the term used for the intervention they described (DH or alternative), the country in which the study was taking place, level at which the intervention was implemented (frontline, management, research), and ticked whether there was a conceptual model, framework, diagram or process description of DH and health management decision-making. This detailed mapping of study characteristics was useful for informing sampling options for Objectives 2 and 3.

#### Sampling of studies

A scoping review aims to map the literature on a particular topic rather than to provide an exhaustive explanation of a particular phenomenon of interest [19, 22]. Thus, the number of included studies was expected to be high in the scoping reviews. To manage the high numbers for a scoping review such as this one (where the aim was to provide definitions and concepts) it was necessary to make use of a qualitative sampling approach. A qualitative sampling approach for this review aimed for variation and depth rather than an exhaustive sample; reviewing too large a number of studies can impair the quality of the analysis and synthesis [23]. We used two types of purposive sampling techniques called maximum variation sampling and theoretical sampling [24]. These techniques were used to identify both the range, variation and similarities or differences in definitions and concepts and intervention descriptions (as per Objective 2) and to provide a rich synthesis of explanations of causal relationships between DH and health management decision-making (as per Objective 3). For Objective 1, we did not apply a sampling strategy. Thus, we included all the studies that at a minimum provided a definition or description of a DH intervention.

### Data extraction

**Fig. 1:**
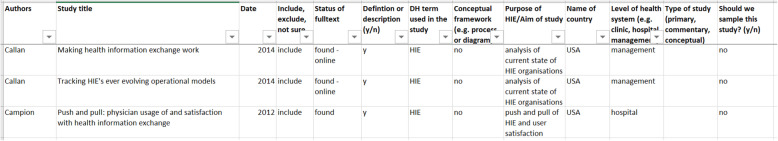
Extract of the Excel data extraction form

BS extracted data for Objective 1 from all the included studies (*n* = 181). AH independently extracted data from 81 (45%) of included studies to verify data extraction done by the first reviewer. We used an MS Excel spreadsheet for data extraction as presented in Fig. 1. AH and BS extracted a few studies before clarifying the items in the spreadsheet. Once data extraction was complete, the reviewers were able to filter according to the individual items extracted to synthesise and compare studies. Given the objectives of the scoping review, we did not extract any information relevant for conducting risk of bias or quality assessment. Not conducting risk of bias or quality assessment is consistent with scoping reviews of similar aims and methodological approaches [19, 22, 25].

### Data synthesis: collating, summarising and reporting findings

The first reviewer (BS) conducted data analysis using manual coding and the filter option in MS Excel. Another reviewer (NL) reviewed the data analysis work on an ongoing basis as an additional quality check. For Objective 1, we conducted a numerical analysis to provide an overview of the characteristics of all the included studies. For Objective 2, we conducted a qualitative analysis to provide a narrative synthesis of the different DH definitions and concepts, and to identify different components or activities that are considered part of the DH processes. For Objective 3, we reviewed data related to intentions, suggestions and or explanations of how DH may lead to improved health management decision-making. We extracted and analysed data relevant to Objective 2 and 3 at the same time. We first created a list of all the different terms used to describe DH interventions and then compared definitions across alternative terms by looking for similarities or differences in the definitions or descriptions of DH interventions. We then coded key components, processes and outcomes of DH interventions and the factors reported as important in the relationship between DH and health management decision-making.

The findings are structured according to three themes matching the three study objectives: an overview of the key characteristics of included studies, alternative terms and definitions of DH, and a narrative synthesis of the relationship between DH and health management decision-making.

### Reflexivity

Throughout the review, the authors were aware of their own positions and reflected on how these could influence the study design, search strategy, inclusion decisions, data extraction, analysis, and synthesis, and interpretation of the findings [23]. The review authors are trained in anthropology, epidemiology, health systems, and evidence synthesis research. The first author was involved in participant observation of an innovative DH project in the Western Cape Department of Health in South Africa as part of her doctoral research where she grappled with questions that informed the objectives of this review. Three of the authors (BS, AH and NL) were involved in a Cochrane systematic review on RHIS interventions when this scoping review was conceptualised, so they were familiar with some of the health information literature (HIS) literature and had some appreciation for the conceptual and methodological complexities of studying the field of health information management. This experience informed the way the first author developed the search strategy. She used an iterative approach to narrow down the search as much as possible because of her prior knowledge that it was difficult to balance sensitivity and specificity when developing a search strategy for HIS literature that is often multi-disciplinary in nature.

## Results

### Results of the search

**Fig. 2:**
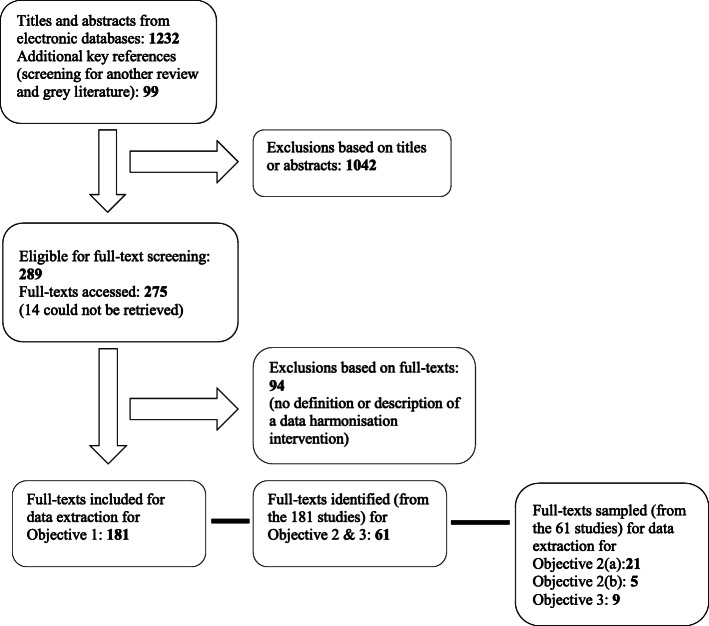
PRISMA diagram of eligible studies

Figure 2 shows a PRISMA diagram of the search results. We screened a total of 1331 records;1232 titles and abstracts identified from searching three electronic databases, and 99 from screening for a Cochrane systematic review assessing the effectiveness of RHIS interventions on health systems management [26] and grey literature. Almost a quarter (289 of 1331) were deemed potentially eligible for full-text screening. We accessed full-texts for 275 studies and of those, 181 were included in the scoping review for Objective 1. We excluded 94 full-text articles because they did not meet the minimum criteria; that is, provide a definition or description of a DH intervention or activity. We sampled 61 studies from the 181 for Objective 2 and 3. We arrived at 61 studies by including all reviews (systematic or literature reviews) and all studies (irrespective of the type of study), that also had a process description, conceptual framework or theory of a DH intervention (that is, in addition to the minimum criteria for Objective 1).

### An overview of key characteristics of data harmonisation studies

A total of 181 studies were included into this scoping review for Objective 1 (see Table 1). Given the high number of included studies, we decided to only map the following key characteristics of those studies: first author, date, type of study, intervention term (DH or alternative), country and level of the health care system. Most included studies (126 of 181) were primary studies assessing various aspects of developing and implementing DH interventions (quantitative studies *n* = 86) or patient, providers or stakeholders’ perspectives (qualitative studies *n* = 34) or a combination of both (mixed methods studies *n* = 6).

**Table 1:** Characteristics of included studies (*n* = 181)

Study name	Date	Type of study	Intervention term	Country	Level of the health care system
**Commentary**					
Burris	2017	Commentary	HIE	USA	Frontline: hospitals
Figge	2010	Commentary	HIE	USA	Management
McIlwain	2009	Commentary	HIE	USA	Management
Murphy	2010	Commentary	HIE	USA	Management
Overhage	2007	Commentary	HIE	USA	Management
Rudin	2010	Commentary	HIE	USA	Frontline: workers
**Conceptual**					
Boyd	2014	Conceptual	RL	Australia	Research
Carr	2013	Conceptual	HIE	USA	Frontline: hospitals
Cimino	2014	Conceptual	HIE	USA	Management
Deas	2012	Conceptual	HIE	USA	Management
Del Fiol	2015	Conceptual	HIE	USA	Frontline: prisons, hospitals
Dimitropoulos	2009	Conceptual	HIE	USA	Management
Downs	2010	Conceptual	HIE	USA	Management
Feldman	2017	Conceptual	HIE	USA	Management
Frisse	2010	Conceptual	HIE	USA	Frontline: patients, workers
Frisse	2008	Conceptual	HIE	USA	Frontline: organisations
Frohlich	2007	Conceptual	HIE	USA	Management
Godlove	2015	Conceptual	HIE	USA	Frontline: patients
Greene	2016	Conceptual	HIE	USA	Management
Grossman	2008	Conceptual	HIE	USA	Management
Haarbrandt	2016	Conceptual	DW	USA	Management
Hu	2007	Conceptual	DS	USA	Management
Jones	2012	Conceptual	DS	USA	Management
Kuperman	2013	Conceptual	HIE	USA	Management
Langabeer	2016	Conceptual	HIE	USA	Management
Liu	2011	Conceptual	HIE	China	Management
McDonald	2009	Conceptual	HIE	USA	Management
McMurray	2015	Conceptual	HIE	USA	Management
Miller	2014	Conceptual	HIE	USA	Frontline: hospitals
Nelson	2016	Conceptual	HIE	USA	Frontline: prisons, hospitals
Politi	2014	Conceptual	HIE	n/a	Management
Ranade-Kharkar	2014	Conceptual	HIE	USA	Management
Shapiro	2016	Conceptual	HIE	USA	Frontline: workers, organisations
Shapiro	2006	Conceptual	HIE	USA	Management
Thorn	2013	Conceptual	HIE	USA	Frontline: health care workers
Thorn	2014	Conceptual	HIE	USA	Frontline: health care workers
Vest	2010	Conceptual	HIE	USA	Management
Williams	2012	Conceptual	HIE	USA	Management
Yaraghi	2014	Conceptual	HIE	USA	Management
Zafar	2007	Conceptual	HIE	USA	Management
Zaidan	2015	Conceptual	HIE	Malaysia	Management
**Primary studies**					
Abramson	2012	Primary, quantitative	EHR, HIE	USA	Frontline, hospitals
Adjerid	2011	Primary, quantitative	HIE	USA	Management, states
Adler-Milstein	2011	Primary, quantitative	HIE	USA	Frontline: organisations
Adler-Milstein	2013	Primary, quantitative	HIE	USA	Management, organisations
Adler-Milstein	2016	Primary, quantitative	HIE	USA	Management
Alexander	2016	Primary, qualitative	HIE	USA	Frontline, health care workers
Alexander	2015	Primary, qualitative	HIE	USA	Frontline, health care workers
Ancker	2012	Primary, quantitative	HIE	USA	Frontline, consumers
Bahous	2016	Primary, quantitative	HIE	Israel	Frontline, hospital
Bailey	2013	Primary, quantitative	HIE	USA	Frontline: hospital
Ben-Assuli	2013	Primary, quantitative	HIE	USA	Frontline: hospitals
Boockvar	2017	Primary, quantitative	HIE	USA	Frontline: hospital
Butler	2014	Primary, qualitative	HIE	USA	Frontline: prisons, communities
Campion	2012	Primary, quantitative	HIE	USA	Frontline: health care workers
Campion	2013	Primary, quantitative	HIE	USA	Frontline: communities
Campion	2013	Primary, quantitative	HIE	USA	Frontline: clinics, hospitals
Campion	2014	Primary, quantitative	DE	USA	Frontline: organisations
Carr	2014	Primary, quantitative	HIE	USA	Frontline: hospitals
Carr	2016	Primary, quantitative	HIE	USA	Frontline: hospitals
Cochran	2015	Primary, qualitative	HIE	USA	Frontline: clinics, communities
Cross	2016	Primary, qualitative	HIE	USA	Management, organisations
Dalan	2010	Primary, qualitative	DM	USA	Research
Dimitropoulos	2011	Primary, quantitative	HIE	USA	Frontline: consumers
Dixon	2013	Primary, quantitative	HIE	USA	Frontline: hospitals
Dixon	2011	Primary, quantitative	HIE	USA	Frontline: laboratories
Downing	2017	Primary, quantitative	HIE	USA	Management: policy
Dullabh	2013	Primary, qualitative	HIE	USA	Management: organisations
Elysee	2017	Primary, quantitative	HIE, IO	USA	Frontline: hospitals
Foldy	2007	Primary, quantitative	HIE	USA	Management: organisations
Fontaine	2010	Primary, qualitative	HIE	USA	Frontline: primary health care
French	2016	Primary, quantitative	HIE	USA	Management: organisations
Fricton	2008	Primary, quantitative	HIE	USA	Frontline: patients, workers
Frisse	2012	Primary, quantitative	HIE	USA	Frontline: organisations
Furukawa	2013	Primary, quantitative	HIE	USA	Frontline: hospitals
Furukawa	2014	Primary, quantitative	HIE	USA	Frontline: health care workers
Gadd	2011	Primary, quantitative	HIE	USA	Frontline: health care workers
Garg	2014	Primary, quantitative	HIE	USA	Frontline: hospitals
Gill	2001	Primary, quantitative	DL	South Africa	Frontline: patients, disease
Grinspan	2013	Primary, quantitative	HIE	USA	Frontline: patients
Grinspan	2014	Primary, quantitative	HIE	USA	Frontline: health care workers
Grinspan	2015	Primary, quantitative	HIE	USA	Frontline: patients
Hassol	2014	Primary, quantitative	HIE	USA	Frontline: health care workers
Herwehe	2012	Primary, quantitative	HIE	USA	Frontline: health care workers
Hincapie	2011	Primary, qualitative	HIE	USA	Frontline: health care workers
Holman	2008	Primary, quantitative	DL	USA	Frontline: organisations, research
Hypponen	2014	Primary, quantitative	HIE	Finland	Frontline: health care workers
Ji	2017	Primary, quantitative	HIE	Korea	Frontline: hospitals
Johnson	2011	Primary, mixed	HIE	USA	Frontline: hospitals
Jung	2015	Primary, quantitative	HIE	USA	Frontline: hospitals
Kaelber	2013	Primary, quantitative	HIE	USA	Frontline: hospitals
Kierkegaard	2014	Primary, qualitative	HIE	USA	Frontline: health care workers
Kierkegaard	2014	Primary, qualitative	HIE	USA	Management
Kim	2012	Primary, qualitative	HIE	Korea	Management
Knaup	2006	Primary. quantitative	DS	Germany	Frontline: hospitals
Kralewski	2012	Primary, qualitative	CIE	USA	Frontline: organisations, workers
Laborde	2011	Primary, quantitative	HIE	USA	Frontline: hospitals
Lee	2012	Primary, quantitative	HIE	South Korea	Frontline: health care workers
Li	2011	Primary, quantitative	DE	Japan & China	Frontline: organisations
Liu	2010	Primary, qualitative	DH	China	Management
Lobach	2007	Primary, quantitative	HIE	USA	Management
Maenpaa	2011	Primary, quantitative	HIE	Finland	Frontline: hospital
Maiorana	2012	Primary, mixed	HIE	USA	Frontline: workers, disease
Martinez	2015	Primary, quantitative	HIE	USA	Frontline: hospitals
Massoudi	2016	Primary, qualitative	HIE	USA	Frontline: organisations
Mastebroek	2017	Primary, qualitative	HIE	Netherlands	Frontline: patients
Mastebroek	2017	Primary, qualitative	HIE	Netherlands	Frontline: patients
Mastebroek	2016	Primary, quantitative	HIE	Netherlands	Frontline: health care workers
Matsumoto	2017	Primary, qualitative	HIE	USA	Frontline: workers, hospitals
Medford-Davis	2017	Primary, quantitative	HIE	USA	Frontline: patients, hospitals
Mello	2018	Primary, qualitative	HIE	USA	Management: policies
Merrill	2013	Primary, quantitative	HIE	USA	Frontline: managers
Messer	2012	Primary, mixed	HIE	USA	Frontline: clinics, organisations
Miller	2012	Primary, qualitative	HIE	USA	Frontline: consumers, organisations
Miller	2017	Primary, quantitative	HIE	USA	Frontline: disease, workers
Moore	2012	Primary, quantitative	HIE	USA	Frontline: workers, hospitals
Motulsky	2018	Primary, quantitative	HIE	Canada	Frontline: workers
Myers	2012	Primary, qualitative	HIE	USA	Frontline: disease, workers
Obeidat	2014	Primary, quantitative	IE	Jordan	Frontline: hospitals
O’Donnell	2011	Primary, quantitative	HIE	USA	Frontline: workers
Onyile	2013	Primary, quantitative	HIE	USA	Frontline: patients
Opoku-Agyeman	2016	Primary, quantitative	HIE	USA	Frontline: hospitals
Overhage	2017	Primary, quantitative	HIE	USA	Management
Ozkaynak	2013	Primary, qualitative	HIE	USA	Frontline: hospitals, workers
Park	2015	Primary, quantitative	HIE	South Korea	Frontline: clinics, hospitals
Park	2013	Primary, quantitative	HIE	South Korea	Frontline: clinics, hospitals
Patel	2011	Primary, quantitative	HIE	USA	Frontline: clinics, hospitals
Politi	2015	Primary, quantitative	HIE	Israel	Frontline: hospital
Ramos	2016	Primary, mixed	HIE	USA	Frontline: patients
Ramos	2014	Primary, qualitative	HIE	USA	Frontline: patients
Reis	2016	Primary, quantitative	HDE	USA	Frontline: hospital
Richardson	2014	Primary, qualitative	HIE	USA	Frontline: organisations, workers
Ross	2010	Primary, qualitative	HIE	USA	Frontline: clinics
Ross	2013	Primary, quantitative	HIE	USA	Frontline: workers, clinics, hospitals
Rudin	2009	Primary, qualitative	HIE	USA	Frontline: health care workers
Rundall	2016	Primary, qualitative	HIE	USA	Management: policy makers, leaders
Saef	2014	Primary, quantitative	HIE	USA	Frontline: hospitals
Santos	2017	Primary, quantitative	HIE	Brazil	Frontline: clinics, hospitals
Shade	2012	Primary, quantitative	HIE	USA	Frontline: clinics, hospitals
Shank	2012	Primary, quantitative	HIE	USA	Frontline: health care workers
Shapiro	2013	Primary, quantitative	HIE	USA	Frontline: hospitals
Shapiro	2007	Primary, quantitative	HIE	USA	Frontline: health care workers
Sicotte	2010	Primary, qualitative	HIE	Canada	Frontline: workers, hospitals
Sprivulis	2007	Primary, quantitative	HIE	Australia	Frontline: workers, organisations
Squire	2002	Primary, mixed	HIE	USA	Frontline: health care workers
Sridhar	2012	Primary, quantitative	HIE	USA	Frontline: hospital
Thornewill	2011	Primary, mixed	HIE	USA	Frontline: consumers, organisations
Unertl	2012	Primary, qualitative	HIE	USA	Frontline: clinics, hospitals
Vest	2010	Primary, qualitative	HIE	USA	Frontline: hospitals
Vest	2017	Primary, qualitative	HIE	USA	Frontline: consumers, organisations
Vest	2015	Primary, qualitative	HIE	USA	Frontline: consumers, organisations
Vest	2013	Primary, qualitative	HIE	USA	Management: policy makers
Vest	2009	Primary, quantitative	HIE		Frontline: workers, patients
Vest	2017	Primary, quantitative	HIE	USA	Frontline: consumers, organisations
Vest	2011	Primary, quantitative	HIE	USA	Frontline: patients, hospitals
Vest	2014	Primary, quantitative	HIE	USA	Frontline: patients, hospitals
Vest	2014	Primary, quantitative	HIE	USA	Frontline: hospitals
Vest	2015	Primary, quantitative	HIE	USA	Frontline: hospitals
Vreeman	2008	Primary, quantitative	HIE	USA	Frontline: laboratory, radiology
Wen	2010	Primary, quantitative	HIE	USA	Frontline: patients
Winden	2014	Primary, quantitative	HIE	USA	Frontline: clinical care
Wright	2010	Primary, quantitative	HIE	USA	Frontline: health care workers
Yeager	2014	Primary, qualitative	HIE	USA	Frontline: consumers
Yeaman	2015	Primary, quantitative	HIE	USA	Frontline: hospital
Zech	2015	Primary, quantitative	HIE	USA	Frontline: patients, organisations
Zech	2016	Primary, quantitative	HIE	USA	Frontline: patients, organisations
Zhu	2010	Primary quantitative	HIE	USA	Research
**Study protocol**					
Dixon	2013	Protocol, mixed	HIE	USA	Frontline: organisations
**Reviews**					
Esmaeilzadeh	2016	Review	HIE	n/a	Management: policy
Esmaeilzadeh	2017	Review	HIE	n/a	Frontline: patients
Fontaine	2010	Review	HIE	n/a	Frontline: primary health care
Hopf	2014	Review	DL	n/a	Frontline: health care workers
Kash	2017	Review	HIE	n/a	Frontline: hospitals
Mastebroek	2014	Review	HIE	USA	Frontline: disease, workers
Parker	2016	Review	HIE	USA	Research
Rahurkar	2015	Review	HIE	n/a	Frontline: hospital
Rudin	2014	Review	HIE	USA	Frontline: clinical care
Sadoughi	2018	Review	HIE	n/a	Management
Vest	2012	Review	HIE	n/a	Management
Dixon	2010	Review	HIE	USA	Research
Akhlaq	2016	Review	HIE	LMICs	Management, countries

Of the 181 included studies, 9 were not country specific (these were global reviews), 151 were from the USA and the rest were from other countries (specifically Australia, Brazil, Canada, China, Finland, Germany, Israel, Japan, Jordan, Korea, Malaysia, Netherlands, South Africa and South Korea). In terms of the level of the health care system, 128 studies were on a DH intervention or activity that was concerned with the frontline level (health service providers), 48 studies were concerned with health system factors or policy-related activities at the managerial level, and 5 studies focused on DH interventions specifically for research purposes. Most studies (92%) used the term health information exchange (HIE), while the remaining studies (8%) used a variety of terms to describe various DH interventions and activities, specifically, record linkage, data mining, data linkage, data warehousing, data sharing and data harmonisation.

### Definitions, components and processes of data harmonisation

We first discuss the alternative terms and definitions of DH and then we summarise key components and processes of DH using studies sampled from the 61 studies identified for Objective 2 and 3. Table 2 presents identifying details of the 61 studies; that is, the type of study design, the intervention terms, the country, the level of the health care system and the purpose of the study (see Table 2). These studies were concerned with the challenges and opportunities of DH, the barriers and facilitators of DH, the various factors affecting DH (such as technical and financial factors), the outcomes of DH (such as patient safety and quality of care), and privacy and security issues of patient information.

**Table 2:** Characteristics of sampled studies (*n* = 61)

Study name	Date	Type of study	Intervention term	Country	Level of the health care system	Purpose of the study
Akhlaq	2016	Review, qualitative	HIE	LMICs	Management, countries	Barriers and facilitators of HIE
Boyd Boyd	2014	Conceptual	RL	Australia	Research	Functions of record linkage
Burris	2017	Commentary	HIE	USA	Frontline: hospitals	Benefits of HIE
Campion	2012	Primary, quantitative	HIE	USA	Frontline: health care workers	Push and pull of HIE
Cimino	2014	Conceptual	HIE	USA	Management	Debates around consumer-mediated HIE
Dalan	2010	Conceptual	DM	USA	Management	Possibilities for clinical data mining and research
Dimitropoulos	2009	Conceptual	HIE	USA	Management	Privacy and security of interoperable HIE
Dixon	2010	Review, framework	HIE	USA	Research	Costs, effort and value of HIE
Downing	2017	Primary, quantitative	HIE	USA	Management: policy	Relationship between HIE and organisational HIE policy decisions
Downs	2010	Conceptual	HIE	USA	Management	Improving laboratory services through HIE
Dullabh	2013	Primary, qualitative	HIE	USA	Management: organisations	Experience of HIE implementation
Elysee Elysee	2017	Primary, quantitative	HIE, IO	USA	Frontline: hospitals	Relationship between HIE, interoperability and medication reconciliation
Esmaeilzadeh	2016	Review	HIE	n/a	Management: policy	HIE assimilation and patterns for policy
Esmaeilzadeh	2017	Review	HIE	n/a	Frontline: patients	Patients’ perceptions of HIE
Fontaine	2010	Review	HIE	n/a	Frontline: primary health care	HIE for primary health care practices
Fontaine	2010	Primary, qualitative	HIE	USA	Frontline: primary health care	Barriers and facilitators of HIE in primary care practices
Frisse	2010	Conceptual	HIE	USA	Frontline: patients, workers	Impact of HIE on patient-provider relationships
Gadd	2011	Primary, quantitative	HIE	USA	Frontline: health care workers	Users’ perspectives on the usability of a regional HIE
Gill	2001	Primary, quantitative	DL	South Africa	Frontline: patients, disease	Linkage of non-communicable diseases data
Greene	2016	Conceptual	HIE	USA	Management	Technical and financial aspects of HIE
Grossman	2008	Conceptual	HIE	USA	Management	Barriers to stakeholder participation in HIE
Haarbrandt	2016	Conceptual	DW	USA	Management	Approaches for a clinical data warehouse
Herwehe	2012	Primary, quantitative	HIE	USA	Frontline: health care workers	Implementation of an electronic medical record and HIE
Hincapie	2011	Primary, qualitative	HIE	USA	Frontline: health care workers	Physicians’ opinions of HIE
Hopf	2014	Review	DL	n/a	Frontline: health care workers	Healthcare professionals’ views of linking routinely collected data
Hu	2007	Conceptual	DS	USA	Management	Challenges in implementing an infectious disease information sharing and analysis system
Hypponen	2014	Primary, quantitative	HIE	Finland	Frontline: health care workers	User experiences with different regional HIE
Ji	2017	Primary, quantitative	HIE	Korea	Frontline: hospitals	Technology and policy changes for HIE
Jones	2012	Conceptual	DS	USA	Management	An overview of electronic data sharing
Kash	2017	Review	HIE	n/a	Frontline: hospitals	Hospital readmission reduction and the role of HIE
Kierkegaard	2014	Primary, qualitative	HIE	USA	Frontline: health care workers	Applications of HIE information to public health practice
Kierkegaard	2014	Primary, qualitative	HIE	USA	Management	Health practitioners’ needs and HIE
Kuperman	2013	Conceptual	HIE	USA	Management	Potential unintended consequences of HIE
Liu	2010	Primary, qualitative	DH	China	Management	Defining data elements for HIE
Maiorana	2012	Primary, mixed	HIE	USA	Frontline: workers, disease	Trust, confidentiality and acceptability of sharing HIV data for HIE
Massoudi	2016	Primary, qualitative	HIE	USA	Frontline: organisations	HIE for clinical quality measures
Mastebroek	2014	Review	HIE	USA	Frontline: disease, workers	HIE in general care practice for people with disabilities
Mastebroek	2016	Primary, quantitative	HIE	Netherlands	Frontline: health care workers	Priority setting and feasibility of HIE
Mastebroek	2017	Primary, qualitative	HIE	Netherlands	Frontline: patients	Experiences of people with intellectual disabilities in HIE
Matsumoto	2017	Primary, qualitative	HIE	USA	Frontline: workers, hospitals	HIE in managing hospital services
Parker	2016	Review	HIE	USA	Research	The use of HIE in supporting clinical research
Politi	2014	Conceptual	HIE	n/a	Management	Use patterns of HIE
Rahurkar	2015	Review	HIE	n/a	Frontline: hospital	Impact of HIE on cost, use and quality of care
Ramos	2016	Primary, mixed	HIE	USA	Frontline: patients	HIE consent process in an HIV clinic
Ranade-Kharkar	2014	Conceptual	HIE	USA	Management	Improving data quality integrity through HIE
Ross	2010	Primary, qualitative	HIE	USA	Frontline: clinics	Motivators, barriers, and potential facilitators of adoption of HIE
Rudin	2014	Review	HIE	USA	Frontline: clinical care	Use and effect of HIE on clinical care
Vest	2016	Primary, qualitative	HIE	USA	Management: policy makers, leaders	Information-sharing needs and HIE
Sadoughi	2018	Review	HIE	n/a	Management	Quality and cost-effectiveness, and the rates of HIE adoption and participation
Santos	2017	Primary, quantitative	HIE	Brazil	Frontline: clinics, hospitals	HIE for continuity of maternal and neonatal care
Shade	2012	Primary, quantitative	HIE	USA	Frontline: clinics, hospitals	HIE for quality and continuity of HIV care
Shapiro	2016	Conceptual	HIE	USA	Frontline: workers, organisations	HIE in emergency medicine
Shapiro	2006	Conceptual	HIE	USA	Management	Approaches to patient HIE and their impact on emergency medicine
Vest	2012	Review	HIE	n/a	Management	National and international approaches of health information exchange
Vest	2015	Primary, qualitative	HIE	USA	Frontline: consumers, organisations	HIE to change cost and utilisation outcomes
Vest	2010	Conceptual	HIE	USA	Management	Challenges and strategies for HIE
Williams	2012	Conceptual	HIE	USA	Management	Strategies to advance HIE
Yaraghi	2014	Conceptual	HIE	USA	Management	Professional and geographical network effects on HIE growth
Yeager	2014	Primary, qualitative	HIE	USA	Frontline: consumers	Factors related to HIE participation and use
Zaidan	2015	Conceptual	HIE	Malaysia	Management	Security framework for nationwide HIE
Zhu	2010	Primary quantitative	HIE	USA	Research	Facilitating clinical research through HIE

#### Alternative terms and definitions of data harmonisation

For Objective 2 (a), we describe alternative terms and definitions of DH. We sampled 21 studies from the 61 studies identified for Objective 2 and 3. The alternative terms and definitions are summarised in Table 3. During data analysis we realised that most studies (53 of 61) used term ‘health information exchange’, with similar definitions. We sample 13 of the 53 studies to contribute to the composite HIE definition in the table. These 13 studies were chosen to represent the term HIE because they were review studies and we assumed that reviews provided synthesised definitions of interventions. Using maximum variation sampling, we included 8 more studies (21 studies in total), because they provided a range of different terms for DH activities, besides the term HIE.

**Table 3:** Alternative terms and definitions of data harmonisation interventions

Liu 2010 [1]	**Data harmonisation** is the process of integrating life-long health data of a person that are distributed in inhomogeneous information systems through identifying, reviewing, matching, redefining and standardising information. This process involves two steps. Firstly, identifying whether all the information necessary for a single electronic platform is available in existing systems, where the information is, and how the information is defined and formatted. And secondly, to make the heterogeneous information recorded by various systems consistent or at least comparable with one another by reviewing, matching, redefining and standardising each data item.
Boyd 2014 [16]	**Record linkage** is the process of bringing together data relating to the same individual from within and between different datasets. When a unique person-based identifier exists, linkage can be achieved by simply merging datasets on the identifier. However, when a person-based identifier does not exist, then some other form of data matching or record linkage is required for integrating data.
Gill 2001 Hopf 2014	**Data linkage** can be used to construct a register for a specific geographic area and disease (for example, a district non-communicable disease register). Linkage of routine datasets by unique patient identifiers can provide an opportunity for identifying adverse drug reactions and tracking exposed individuals in real time. Routine data linkage can also enable the creation of exposure cohorts to monitor long-term outcomes and enable a more efficient screening for adverse drug reactions due to an ever-increasing data pool.
Haarbrandt 2016 [28]	**Data warehousing** is the process of establishing specialised databases by integrating information systems (the authors specifically referred to hospital information systems) to facilitate secondary use of data. Clinical data warehouses are generally built on one of two predominant architectural paradigms: either, data is directly extracted, transformed and loaded from applications systems and databases into a data mart (an integrated view over a defined subject), or it is stored in a centralised data repository from which data marts can be established. Both approaches rely on a process to extract data from sources, transform it appropriately and to load it (or copy it) to a specific database.
Hu et al., 2007 [17] Jones 2012	**Data sharing** is based on the need for a more robust method for defining and sharing expected and actual patient outcomes. It must leverage existing informatics tools since a great deal of patient-specific information is already available in medical record systems and billing and administrative systems. One type of data sharing system is an infectious disease informatics (IDI) system. An IDI system should encompass sophisticated algorithms for the automatic detection of emerging disease patterns and the identification of probable threats or events. It should also have advanced computational models that overlay health data for spatial–temporal analysis to support public health professionals’ analysis tasks.
Elysee 2017 [29]	**Data interoperability** is one of two functionalities of an advanced electronic health record. The first function is health information exchange, which is the ability to electronically share patient-level information among unaffiliated providers across organisational boundaries. The second function is interoperability, which is the ability to produce standardised patient-level health information that can be integrated into unaffiliated health care providers’ electronic health records.
Akhlaq 2016 [15] Dixon 2010 [33]Esmaeilzadeh 2016 [34] Esmaeilzadeh 2017 [35] Fontaine 2010 [36] Hopf 2014 [38] Kash 2017 [39] Mastebroek 2014 [27] Parker 2016 [42] Rahurkar 2015 [44] Rudin 2014 [45] Sadoughi 2018 [46] Vest 2012 [48]	**Health information exchange (HIE)** is a type of health information technology (HIT) intervention. It involves the electronic mobilisation of clinical and administrative data or information within or across data repositories or organisations in a community or region, between various systems as per recognised standards. This is to ensure that the HIE maintains the authenticity and accuracy of the information being exchanged, thereby enabling stakeholders to make informed decisions to enhance healthcare quality and delivery of patients and populations. Sharing clinical data can potentially improve patient safety, care coordination, quality of care and efficiency, facilitate public health efforts and reduce mortality and healthcare costs. Lastly, HIE involves multi-stakeholder organisations that oversee the business, operational and legal issues involved in the exchange of information.

Where multiple studies used a similar definition, the review authors synthesised the data from similar definitions into the composite definition for each term, as presented in this table

There is overlap between the terms and definitions. Definitions for data harmonisation, record linkage and data warehousing explicitly state that these interventions involve a process of having to integrate different or ‘homogeneous’ databases or information systems. Data linkage and record linkage both focus on ‘linkage’ as a core activity in combining different databases using a unique patient identifier. HIE is described as a key outcome of data interoperability, that is, where the focus is on technical linkage of different electronic data bases. Data sharing, where the focus is on data accessibility and use, is described as a key outcome of HIE.

Based on the literature, we identified elements found in the various definitions of data harmonisation. DH is considered a multi-step process with a range of activities (such as identifying, reviewing, matching, redefining and standardising information). Data harmonisation interventions rely on interoperability between databases and systems which means copying standardised patient-level data into a separate repository. Data linkage and record linkage are activities of a broader intervention (data harmonisation), using mechanisms (such as unique patient identifiers) for integrating large datasets. Data warehousing is concerned with extracting, transforming and loading large datasets using information technology (IT) platforms, application systems and data displays (data marts or data dashboards). Data sharing (through the accessing and exchanging electronic health information), can be considered an outcome of HIE interventions. The aim of these interventions is to integrate and make data accessible across different platforms (such as clinical and financial systems), and to allow for the sharing of this data across the patient care trajectory. The ultimate aim of DH, it would seem, is to improve patient outcomes, coordination of health services, quality of care and efficiency and facilitate public health interventions.

In reviewing the definitions, we identified nine characteristics of DH. No single study included all these characteristics, and there are no specific factors such as study design, country or level of the health care system associated with the definitions. DH is characterised by the following characteristics:

- Any type of DH intervention or activity is a ***process of multiple steps*** involving both technical and social processes.
- The goal of a DH intervention or activity is to ***integrate, harmonise and bring together*** different electronic databases into useable formats.
- There are at least ***two or more databases*** involved in any DH intervention or activity.
- A data harmonisation intervention or activity involves ***electronic data*** (no reference is made to data found in paper-based sources).
- Data harmonisation occurs when there is an increasing availability of electronic data that can be pooled together using ***unique patient identifiers***.
- ***Different types of data*** can be linked and shared such as individual patient clinical, pharmacy and laboratory data, health care utilisation and cost data, and personnel-related data.
- Electronic data required for DH processes can be found within and across ***different departments and institutions at facility, district, regional and national levels***.
- A data harmonisation process consists of ***different types of technical activities*** such as identifying, reviewing, matching, defining, redefining, standardising, merging, linking, merging and formatting data.
- DH interventions or activities are defined according to a ***specific scope and purpose*** such as disease surveillance, monitoring of long-term outcomes, screening for adverse events, geographic area, secondary data use and data display mechanisms (data marts or dashboards).

#### Components and processes of data harmonisation

To synthesise key components and processes of DH interventions (Objective 2(b)) we sampled 5 from the 61 studies identified for Objective 2 and 3. We selected 5 studies [16, 17, 29–31] based on the conceptual descriptions and visual illustrations of their DH interventions (See Table 4).

**Table 4:**
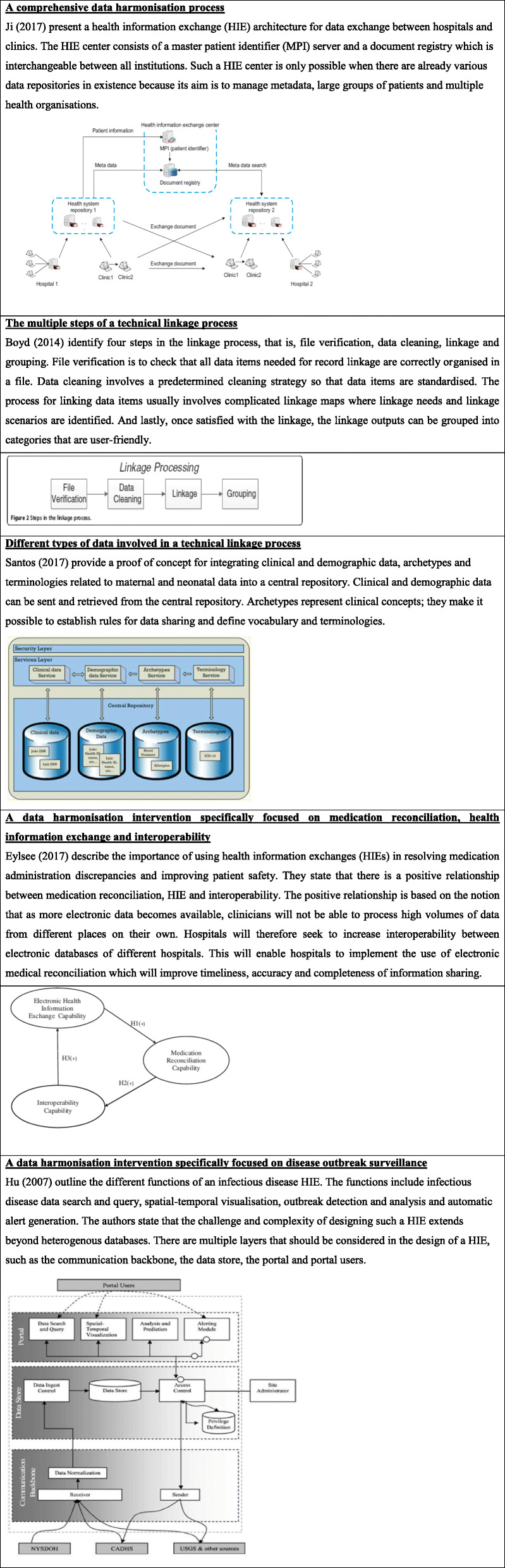
Concepts of data harmonisation interventions and processes

The table presents the different conceptual models of data harmonisation and the review authors provide a summary of how key components and processes were described by the authors of these models

The conceptual description by [30], comes closest to a comprehensive conceptual model of a DH intervention, illustrating different types of data, different levels of the health care system (e.g. clinics and hospitals), the multiple processes of exchanging data, the multiple directions in exchange of data, and the key role of the unique patient identifier in enabling the DH process [30]. In the next model, Boyd et al. [16] and Santos et al. [31] both lay out the technical processes involved in the linkage process of different databases, but Santos et al. specifically focuses on linking data required for individual patient clinical management into a central repository. Lastly, Elysee et al. [29] and Hu et al. [17] describe DH interventions with different purposes, that is, medication reconciliation and disease outbreak surveillance respectively.

These conceptual models of DH interventions and activities highlight that there are various steps involved in the integration of databases and in the transformation of data into useable formats. Integrating databases means bringing together data of the same individual from within and between different electronic databases, through various activities involving identifying, reviewing, matching, redefining and standardising data [1, 16]. Once data is harmonised, it can be categorised by various criteria of interest, such as geographic area or disease or patient population, and transformed into different formats such as graphs, tables or dashboards to make it easier for users to access and use the information [28]. There may be different ways that the data is harmonised; in some studies, DH is described as a linear and one-directional process, while other studies described it as an iterative and multi-directional process.

### The relationship between data harmonisation and health management decision-making

We sampled 9 studies from the 61 studies (identified for Objective 2 and 3) that provide an explanation of the relationship between DH and health management decision-making. These 9 studies were selected because they referred to the intended benefit, or directly referred to the relationship between DH and health management decision-making. We present extracts of explanations of the relationship in Table 5**.** According to Eylsee et al. [29] (the study providing the most detail), there is a positive relationship between increased availability of electronic data sets and the ability of clinicians to deal with high volumes of data. This necessitates interoperability between electronic databases at different hospitals, to improve timeliness, accuracy, and completeness of information sharing. According to Ji, Boyd, Santos and Hu the main benefit of DH is health management decision-making, including clinical decision-making [16, 17, 30, 31]. Across the studies, there is agreement that DH interventions make it possible for health providers to use data over time and across organisations to support clinical management decision-making. There is acknowledgement that DH interventions were sometimes unable to deal effectively with inconsistencies, incompleteness, and poor quality of data.

**Table 5:** The relationship between DH interventions and health management decision-making

Cimino 2014 [21]	“Data completeness: A promise of HIEs is to use consolidated information over time and across providers to improve **medical decision-making for the patient**. When presenting a medical timeline for a patient, how does a provider know whether the HIE presentation of history is missing information? The consequences to patients can be devastating.”
Downs 2010 [32]	“… community-based approach to establish a common pathway based on common data standards to facilitate the incorporation of interoperable, clinically useful genetic or genomic information and analytical tools into EHRs to **support clinical decision-making for the clinician and consumer**.”
Grossman 2008 [37]	“… the exchanges going beyond core clinical data exchange activities that give physicians access to data at the point of care to offering **physicians clinical decision support**, **reminders and other quality improvement tools aimed at individual patients.”**
Kuperman 2013 [40]	“Ideally, a physician would have access to complete, accurate and timely patient data to **support optimal decision making**. Health information exchange capabilities will reduce the extent of data fragmentation but will not eliminate it entirely.”
Politi 2014 [41]	“In this scenario, an HIE system is likely to have a significant impact on **clinical decision making** if information is readily accessible; the need for rapid decisions might render the scrutiny of an HIE system impractical.”
Vest 2010 [43]	“The anticipated benefits of more data to inform **physician decision making**, sparing patients of needless tests, helping organization identify inappropriately managed patients, and improving the health of the public will only be achieved by HIE that does not exclude providers in an area, limit what data elements are available, or restrict exchange to specific subpopulations.”
Shapiro 2006 [47]	“The goal of a nationwide health information network would be to deliver information to individuals– consumers, patients, and professionals –when and where they need it, so they can use this information to **make informed decisions about health and health care**.”
Vest 2015 [49]	“Improved access to more comprehensive information may **support decision-making**, inform providers of additional medications or allegories, and help avoid repeated or duplicate testing.”
Zaiden 2015 [50]	“Combined with data mining and statistical analysis tools, these repositories of health information can greatly advance medical knowledge, healthcare quality, and **good strategic management**.”

The review authors directly quoted text from the primary studies where a description of the link between data harmonisation and health management decision-making was provided

From the 9 studies, we identified three types of health management decision-making that DH contributed to. These are:

- Clinical decision-making for individual patient clinical management or clinical support and quality improvement tools
- Operational and strategic decision-making for health system managers and policy-makers
- Population-level decision-making for disease surveillance and outbreak management

The first level involves frontline clinicians being able to access their patients’ medical information and treatment data and timelines (datasets of longitudinal, clinically relevant individual-level data) through DH interventions. In these situations, DH can make it easier for frontline clinicians to develop tools for reminding them about patients’ performance in treatment and care services as well as help them improve the quality of health care services. At the operational and strategic decision-making level, DH interventions have the potential to support high-level health managers in decision-making involving a wide network of stakeholders (consumers, patients and professionals). Lastly, disease surveillance and outbreak management decision-making rely on harmonised data to plan, monitor and evaluate population-level interventions.

## Discussion

### Synthesis of findings

This scoping review aimed to provide an overview of the key characteristics of DH studies, identify definitions, alternative terms, components, and processes of DH interventions, and provide explanations of the relationship between DH and health management decision-making. Of the 181 studies that at a minimum provided a definition or description of a DH intervention or activity, 86 were primary quantitative studies, 151 were studies conducted in the USA, and 128 were aimed at improving frontline level health services.

A key finding is that ‘Health information exchange’ or HIE, was the term most frequently used in the literature, especially for studies for the USA. Other terms used were data harmonisation, record linkage, data linkage, data warehousing, data sharing, and data interoperability. Terms like data harmonisation and data warehousing seem to describe a more comprehensive approach to DH interventions (involving both data production and data utilisation aspects), whereas terms like record and data linkage described specific activities within health information exchange. The term data interoperability focuses on the technical aspects that allows for different electronic databases to be linked and for data to be integrated, which allows for synthesis and analysis of health information. Even though different studies used different terms, there was consensus that DH is a useful tool for health management decision making and can support improvements in patient and health system outcomes.

We identified nine characteristics of DH interventions and activities. Using these nine characteristics, DH can be summarised as a process that aims to integrate two or more electronic databases, it involves different types of data captured within and across various institutions at different health care system levels, and varying activities are required to pool together data using unique patient identifiers for the purpose of providing information support for health management decision-making. The review identified three types of health management decision-making that DH contributed to: (a) clinical decision-making for individual patient management, clinical support and quality improvement tools; (b) operational and strategic decision-making for health system managers and policy-makers; and (c) population-level decision-making for disease surveillance and outbreak management.

**Fig. 3:**
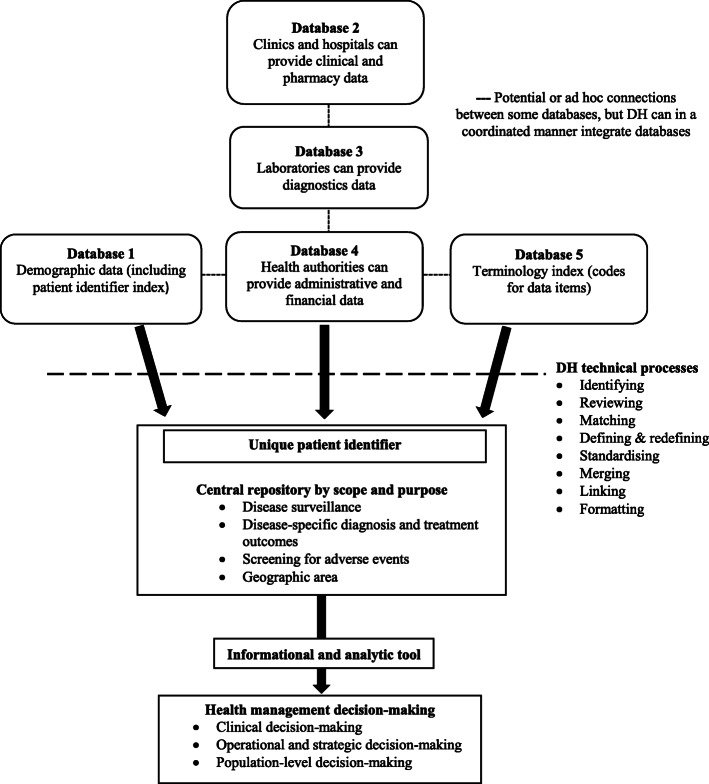
A concept map of data harmonisation and its relationship to health management decision-making

Drawing on the definitions and the conceptual models of DH identified in this review, we developed a concept map (see Fig. 3) to explain how different aspects of DH interventions and activities work together to support health management decision-making. The concept map consists of different types of databases (1 to 5) containing different types of data such as demographic, clinical, pharmacy, laboratory, administrative and financial, and terminology data. A technical process involving different types of activities (such as matching, merging and linking) takes place to integrate the different types of data using a unique patient identifier. The central repository, where the data is harmonised, is defined according to specific criteria such as a geographic area or disease outcomes. The data kept in the repository should be accessible to data users, who can then use this harmonised data as an information and analytic tool to support health management decision-making for clinical, operational, strategic, and or population-level decision-making.

### Study limitations

There are two main differences between the published protocol and this scoping review. We did not search the Global Health database as planned; we realised late that none of the reviewers had permissions to access the database and gaining access was not affordable. We did however manage to search at least three electronic databases, as is the convention in reviews [23]. Due to the large volume of studies included for full-text screening, it was not feasible to conduct the full text screening in duplicate as planned. The first reviewer (BS) assessed all full-texts and then the second reviewer (AH) verified the decisions of the first reviewer in a third of the included studies, which allowed for additional quality checks.

There are two main limitations of the review. Firstly, we restricted our literature search to English. We did not have the resources required for reviewing non-English studies. Most studies identified were from the USA, but it is possible that studies from other non-English speaking, high-income countries with extensive electronic health systems (such as France) may have been missed. Secondly, although sampling aimed to identify variety, comprehensiveness and meaningfulness of the definitions and explanations, there is a possibility that due to sampling, we may have missed relevant studies for Objectives 2 and 3.

### Implications for research and practice

There is a need to understand what DH interventions and activities are comprised of in diverse settings and contexts, especially in LMICs. There were fewer studies from LMICs, which may be due to a lower prevalence of electronic health information systems in those settings. Nevertheless, DH interventions hold promise for improving the informational support in LMICs; studies in these contexts could usefully expand the evidence base.

The review highlights the importance of providing detailed descriptions of DH interventions, to allow for better comparisons and to improve the transferability of study results. Additionally, many resources are spent on the technical development of DH projects, with the implicit assumption that this will provide the informational and analytic support for health management decision-making, but this assumption is seldom tested in the research. There is a need for qualitative research on the health system factors of implementing DH and for formative work to inform design of DH interventions. Finally, primary research and evidence synthesis of the experiences of key stakeholders involved (implementers and users of harmonised data) would improve our understanding of the causal mechanisms between data harmonisation and health systems strengthening.

## Conclusion

The review aimed to widen our understanding about the range of definitions, components and processes of DH interventions, and how it can contribute to health management decision-making. Most studies of DH interventions and activities were conducted in high-income settings and used the term ‘health information exchange’. The review described the processes, technical activities, types of data, mechanisms for integrating data, and purpose of the DH interventions. DH interventions contributed to three types of health management decision-making, that is, clinical decision-making, operational and strategic decision-making, and population-level surveillance decision-making. We provided a concept map of the components of DH and make recommendations for future research.

## Data Availability

The datasets used and/or analysed during the current study are available from the corresponding author on reasonable request.

## Abbreviations

DH: Data harmonisation
RHISs: Routine health information systems
LMICs: Low- and middle-income countries
MeSH: Medical Subject Headings
HIS: Health information system
IT: Information technology
HIE: Health information exchange
